# Platelet Mitochondrial DNA Methylation as Epigenetic Biomarker of Short-Term Air Pollution Exposure in Healthy Subjects

**DOI:** 10.3389/fmolb.2021.803488

**Published:** 2022-01-19

**Authors:** Huimin Sun, Yanting Li, Jianzhong Zhang, Teng Shi, Xin Li, Xue Cao, Tao Wang, Nan Kong, Yaxian Pang, Tsendmaa Bold, Yuxin Zheng, Rong Zhang, Jinglong Tang

**Affiliations:** ^1^ Department of Occupational and Environmental Health, School of Public Health, Qingdao University, Qingdao, China; ^2^ Department of Toxicology, School of Public Health, Hebei Medical University, Shijiazhuang, China

**Keywords:** air pollution, short-time exposure, platelet count, platelet mitochondrial methylation, epigenetic biomarker

## Abstract

Air pollution exposure is now considered a growing concern for global public health. RNA or DNA methylation changes caused by air pollution may be related to the development of cardiovascular disease. To investigate the early biomarkers of air pollution exposure, a panel study of eight college students recorded after a business trip from Qingdao to Shijiazhuang and back to Qingdao was performed in this work. The concentration of PM_2.5_, PM_10_, SO_2_, NO_2_, and CO in Shijiazhuang was higher than that in Qingdao during the study period. The platelet count was positively correlated with air pollutants of 0–6 day moving averages (β_PM2.5_ = 88.90; β_PM10_ = 61.83; β_SO2_ = 41.13; β_NO2_ = 57.70; β_CO_ = 62.99, respectively, for an IQR increased). Additionally, internal dose biomarkers 2-OHNa, 1-OHNa, 2-OHFlu, 2,3-OHPhe, and ∑PAHs were also significantly associated with platelet count in participants. Furthermore, PM_2.5_ and PM_10_ are positively linked with methylation of one CpG site at platelet mitochondrial gene *CO2* (PM_2.5_ = 0.47; PM_10_ = 0.25, respectively, for an IQR increase). Both platelet counts and methylation levels returned to their pre-exposure levels after leaving the highly contaminated area. In short, this study investigated the relationship between platelet properties and air pollution exposure, revealing that short-term exposure to air pollution might increase the risk of thrombosis. Our research suggests that platelet count and mitochondrial DNA methylation of *mtCO2* site *2* in platelets from healthy adults may be the novel biomarker for acute exposure to air pollution.

## Introduction

According to the World Health Organization (WHO), air pollution causes approximately 7 million deaths annually (Kuehn, 2014), with cardiovascular illnesses accounting for the majority of these deaths (Brook et al., 2010; Miller and Xu, 2018). Thrombosis is the most prevalent pathological change associated with cardiovascular disease, and it is directly linked to platelets’ occurrence and development. Numerous epidemiological studies have shown that air pollution affects pro-thrombosis, specifically elevating platelet activation markers (Baccarelli et al., 2007; Devlin et al., 2012; Poursafa and Kelishadi, 2010). However, previous epidemiological studies only focused on a few biomarkers of platelet activation (such as sCD40L) and paid less attention to platelet characteristics and DNA methylation changes of platelet mitochondria (Poursafa and Kelishadi, 2010). Therefore, the comprehensive analysis of platelet-related indexes is urgent for finding more sensitive biomarkers.

Mature platelets, 2–4 µm, which survived for 7–10 days after exfoliation from megakaryocytes, act as central hemostasis, and their cross-talk with other blood cells sustains the stability of the circulation system. Moreover, the platelets are involved in different biological processes, including wound healing, angiogenesis, immune regulation, and inflammation, which are all fundamental components of the stress response (Koupenova et al., 2018; van der Meijden and Heemskerk, 2019). Some evidence suggests that particulate matter (PM) in the air causes platelet activation through different pathways, such as endothelial cell damage. Subsequently, a clotting cascade begins as the thrombus expands and platelet levels are released in large quantities (Melchinger et al., 2019). Animal studies have also shown a shift in the balance between platelet activation, coagulation, and fibrinolysis to pro-coagulation and anti-fibrinolysis states after exposure to air pollutants (Dobrakowski et al., 2016; Daiber et al., 2017). Nevertheless, the effect of acute air pollution on platelet changes in healthy people is still controversial (Li et al., 2018a; Lin et al., 2019); furthermore, due to the limitation of methods, there are few studies to explore the epigenetic effects of air pollution on platelet genes in the general population.

Recent studies in environmental epigenetics provide opportunities to understand the mechanistic underpinnings of exposure-related health effects (Martin and Fry, 2018). Epigenetic modifications, including chemical modifications of DNA, RNA, and proteins, are based on changes in gene expression and function levels that are not caused by sequence changes. RNA methylation is regulated by upstream DNA methylation (Zhou et al., 2019). Since platelets have no nucleus, epigenetic regulation of their mitochondrial genome is critical for platelet activation and the development of cardiovascular disease (Byun et al., 2013). Human mitochondrial DNA is a 16.5-kb double-stranded circular, super-coiled, and compact molecule. Moreover, the number of mitochondria is 3–10 times more than that of nuclear DNA, and the mitochondria are particularly vulnerable to oxidative damage due to the absence of histone protection and an inefficient DNA repair system (Matilainen et al., 2017). Platelet mitochondrial DNA methylation appears to be higher in patients with cardiovascular disease, according to evidence (Baccarelli and Byun, 2015). Platelet mitochondrial gene methylation has also been used as a potential biomarker for cardiovascular disease. Despite that, there are still few studies on platelet mitochondrial DNA methylation in healthy adults after acute exposure to air pollution.

Shijiazhuang, located in China’s interior, is one of the most polluted cities in northern China. The main reason is that the terrain is low in the west and high in the east, and the prevailing northwest wind makes it difficult for the air to enter the urban area, which makes it difficult for the air pollutants emitted by the heavy industry in the city to disperse. In contrast, Qingdao is a coastal city with light industrial pollution, and the sea breeze can remove some of the city’s air pollutants. Previous studies by the team have shown that the average annual level of PM_2.5_ in Shijiazhuang’s air is higher than that in Qingdao (ranging from 45 to 220 μg/m^3^ vs. ranging from 22 to 92 μg/m^3^) (Wang et al., 2020b; Guiqin et al., 2020; Geng et al., 2021; Zhang et al., 2021). The difference in air pollution between the two cities provides a natural experimental opportunity to investigate the effects of acute exposure to air pollution on human biomarkers of the platelet. In December 2019, eight college students from Qingdao University went to Shijiazhuang for a six-day business trip. The platelet parameters, including platelet count (PLT), platelet hematocrit (PCT), platelet distribution width (PDW), mean platelet volume (MPV), platelet large cell ratio (P-LCR), and methylation of the CpG site at platelet mitochondrial genes, were evaluated for volunteers exposed to severe air pollution in Shijiazhuang compared to Qingdao before and after a business trip.

## Methods

### Participants and Research Design

Eight healthy young participants from Qingdao University, who had to travel to Shijiazhuang on a six-day business trip for a project, were recruited. Enrollment required each candidate to attend an interview, including a questionnaire on general health and demographic characteristics to ensure that the candidate participants were in a good general state of health of the body. The inclusion criteria were as follows: 1) age >18 years old and 2) body mass index <30 kg/m^2^. Exclusion criteria were as follows: 1) a history of heart disease; 2) asthma symptoms; 3) kidney disease; 4) coagulopathy; and 5) rheumatic disease, or chronic inflammation within the past 6 months. The Institutional Review Board of Qingdao University approved the following study proposal. All research conducted on each participant accepted by themselves through signed an informed consent form after informed of all aspects of the study before starting the study, respectively.

The air quality in Shijiazhuang was seriously polluted compared with that in Qingdao (Li et al., 2017; Li et al., 2019). Blood and urine samples were collected over 3 measurement time points: 1) before departure from Qingdao to Shijiazhuang (QD-before); 2) the first day after return from Shijiazhuang to Qingdao (SJZ); and 3) 1 week after return to Qingdao (QD-after).

### The Collection of Air Quality Status

The air pollution index (API) in both cities was evaluated with various indicators such as PM_2.5_, PM_10_, SO_2_, and other pollutants obtained from the national urban air quality real-time detection data released by the China Environmental Monitoring Center (http://www.cnemc.cn). Thereupon, air quality record time covers the entire process of the scheduled study (December 12, 2019–December 29, 2019). To calculate the average daily concentration of each indicator during the study period, we recorded the 24-h value of the entire study period in real time.

### Sampling of Blood and Urine

Venous blood and morning urine samples were collected from the participants. Participants were asked to fast for 8 h before sample collection to reduce the effect of diet on biomarker concentrations. In total, we had 24 EDTA anticoagulated blood samples and 24 urine samples (we collected samples of each person for 3 periods). While some of the blood was tested for routine blood tests (Qingdao Municipal Hospital), the rest was separated from the plasma and stored at −80°C until platelet mitochondria DNA extraction and pyrosequencing. The platelet parameters involved in this study included platelet count (PLT), platelet hematocrit (PCT), platelet distribution width (PDW), mean platelet volume (MPV), platelet large cell ratio (P-LCR), and methylation of the CpG site at platelet mitochondrial genes. For PAH (polycyclic aromatic hydrocarbon) metabolite assessments, fresh urine samples were collected for the further HPLC-MS/MS evaluation.

### Polycyclic Aromatic Hydrocarbons in Urine

The concentrations of PAH metabolites, including 2-hydroxynaphthalene (2-OHNa), 1-hydroxynaphthalene (1-OHNa), 2,3-hydroxyphenanthrene (2,3-OHPhe), 1-hydroxypyrene (1-OHP), and 2-hydroxyfluorene (2-OHFlu), in urine were used as internal dose biomarkers and determined using the HPLC-MS/MS method by a blind tester to exposure status. The standards were purchased from Chem Service and Sigma-Aldrich. 1 ml of sodium acetate buffer and 20 μL of *β*-glucosidase were mixed into 2 ml of urine sample, and then the mixture was incubated at 37°C for 8 h in the dark. Eight μL internal standard was added into the urine samples, and then we adjusted the pH value to 5. Subsequently, urine with 4 ml dichloromethane was evaporated to dryness using nitrogen before being dissolved with 10 μL HPLC solvent. Finally, a liquid chromatographic system (LC-20AD, Shimaji, Japan) and an integrated triple quadrupole mass spectrometer (ABI3200, Applied Biosystems, United States) were used for the analysis. Urinary creatinine, measured using an ELISA kit (MEIMIAN, China) according to the manufacturer’s instructions, was used to standardize urinary polycyclic aromatic hydrocarbon metabolite concentrations.

### Extraction and Bisulfite DNA Conversion of Mitochondrial DNA in Platelets

To convert mitochondrial DNA into bisulfate for sequencing, plasma samples from 8 participants were used to isolate platelet mtDNA as described before. Briefly, platelet pellets were obtained by centrifugation of 400 μL of plasma samples at 1,400 × g for 15 min at room temperature. To minimize contamination with nuclear DNA, we added 3 μL of DNase I (Solarbio, China, 10 U/μL) and 7 μL deionized water and incubated the samples for 3 h at 37°C. The samples were treated with 20 μL proteinase K at 50°C for 1 h and centrifuged at 1,000 × for 10 min for purification, and then the supernatants were retained, respectively. According to the protocol provided by the EZ DNA methylation^TM^ Kit (Zymo Research, CA, United States), the mitochondrial DNA was extracted and transformed into bisulfite-converted mtDNA.

### PCR and Pyrosequencing

The mitochondrial primer sequences were selected as stated in the references (Baccarelli and Byun, 2015), which designed four assays to interrogate mitochondrial DNA methylation based upon the GeneBank: J01415.2 (L-strand) mitochondrial genome sequence using the Meth Primer program. DNA methylation was measured at two CpG sites within *MT-ATP8* (Forward: TAT​TAA​TTG​GTT​TTT​TAG​GGT​TTA​T; Reverse-bio: CAACAAATCTTTCATATTACTTCC; Seq primer: TATTTATAGTAGGAAT) and *MT-TL1* (Forward: TAG​GGT​TTG​TTA​AGA​TGG​TAG​AGT​T; Reverse-bio: TAG​GGT​TTG​TTA​AGA​TGG​TAG​AGT​T; Seq primer: TAGGGTTTGTTAAGATGGTAGAGTT), three CpG sites within *MT-ND5* (Forward: GAG​ATA​TAT​AAA​TTT​AGA​T TTAAATATTAA; Reverse-bio: TAA​ACA​AAA​AAA​ATA​TAA​TTC​CTA​C), and six CpG sites within *MT-CO2* (Forward: TTT​ATA​GAG​TTG​TTT​TTA​TAT​TAG​GTT​TAA​A; Reverse-bio: ACT​CCA​CAA​ATT​TCA​AAA​CAT​TAA​C; Seq primer: TAAAAATAGATGTAAT). PCR was performed using 1 µL bisulfite-treated mitochondrial DNA and 25 µL Epiarttm_hs Taq Master Mix (Vazyme, China). The amplified target genes (5 µL) were taken, and 2 µL loading buffer was added for DNA gel electrophoresis to verify whether the amplification of the target genes was successful. Amplified mtDNA PCR products (10 µL) were then used for pyrosequencing by using PyroMark Q48 Autoprep (QIAGEN, Valencia, CA, United States).

### Statistic Analysis

The participants’ basic information and air quality were expressed as mean ± SD and otherwise by median and range (IQR) depending on whether they were normally distributed demographic and clinical characteristics. Spearman correlation analysis was carried out for various pollutants. Linear mixed-effects models with a random intercept for each participant were used to estimate the associations between air pollutants and biomarkers. Considering that the skewed distribution of exposure indicators may lead to poor model fit, urinary PAH metabolites (∑PAHs, 2-OHNa, 1-OHNa, 2,3-OHPhe, 1-OHP, and 2-OHFlu) of participants were logarithmic-converted to better fit the normality hypothesis. The least absolute redundant selection operator (LASSO) was used to filter variables into the mixed models. The covariates included age, sex, height, BMI, and smoking status. Hence, the interquartile range (IQR) of API (PM_2.5_, PM_10_, SO_2_, NO_2_, and CO) was calculated based on daily averages over the whole study period. The air pollution metrics entered into the models as continuous variables and each regression coefficient acquired finally provide an estimate of the effect in terms of an interquartile (IQR) increase in each air pollutant exposure. Similar approaches have been taken to assess the effect of PAH with biomarkers. Sensitivity analysis was conducted by excluding a current smoker (*n* = 1) to test the impact of smoking on the biomarker. For an appropriate supplementary approach to adjust the false discovery rate of the model, the Benjamini–Hochberg method was applied in multiple comparisons to avoid Type I errors. All statistical analyses were performed using SAS software, version 9.4 (site 70239492). All reported *p*-values less than 0.05 were defined as statistically significant.

## Results

### Characteristics of Study Subjects

A total of 8 participants were invited to participate in the study (Table 1). There were five (62.5%) males and three (37.5%) females studying for a graduate degree, and their ages were between 24 and 28 years. The overall mean (SD) age of respondents was 24.88 ± 1.30 years. Specifically, participants had an average BMI of 23.27 ± 2.08 kg/m^2^, including two who were overweight (BMI = 25.39 and 25.35), according to the standards of the WHO. One male participant was a current smoker (12.5%). The participants did not receive any medicine and participated in the complete research process.

**TABLE 1:** Demographic characteristics of research subjects.

Demographics	All subjects (*n* = 8)
Age (yr, M ± SD)	24.88 ± 1.30
Weight (kg, M ± SD)	67.25 ± 9.58
Height (cm, M ± SD)	169.50 ± 5.64
BMI (kg/m^2^, M ± SD)	23.27 ± 2.08
Smoking status	
Never smoker (*n*, %)	7, 87.50
Current smoker (*n*, %)	1, 12.50

Definition of abbreviations: BMI, body mass index; M, mean; SD, standard deviation

### Exposure Assessment

**FIGURE 1:**
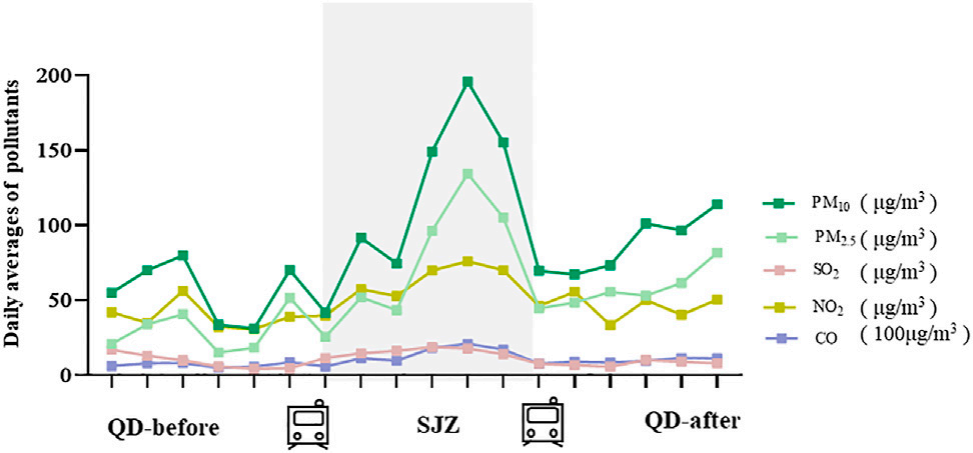
Differences in air quality between Shijiazhuang and Qingdao during the study period. QD-before: 2020.12.12-2020.12.17 Qingdao air quality condition; SJZ: 2020.12.18-2020.12.23 Shijiazhuang air quality condition; QD-after: 2020.12.24-2020.12.29 air quality condition.

The average concentrations of PM_2.5_, PM_10_, SO_2_, NO_2_, and CO were 76.25 μg/m^3^, 118.04 μg/m^3^, 15.95 μg/m^3^, 61.04 μg/m^3^, and 1.39 mg/m^3^ during the period in Shijiazhuang city (Figure 1; Supplementary Table S1). Compared with the QD-before, they were higher by 1.88, 2.39, 2.00, 2.00, and 1.98 times. The mean concentration of PM_2.5_, PM_10_, and NO_2_ in the three periods (Supplementary Table S1) was higher than the recommended limits as stated in the 2005 WHO reports (50 μg/m^3^, 25 μg/m^3^, and 40 μg/m^3^). There were strong correlations between the pollutants during the whole study period (*r* ≥ 0.60) (Supplementary Table S2). For example, the Spearman’s correlation coefficient between PM_2.5_ and PM_10_ was 0.98, and that of PM_2.5_ and CO was 0.97.

**FIGURE 2:**
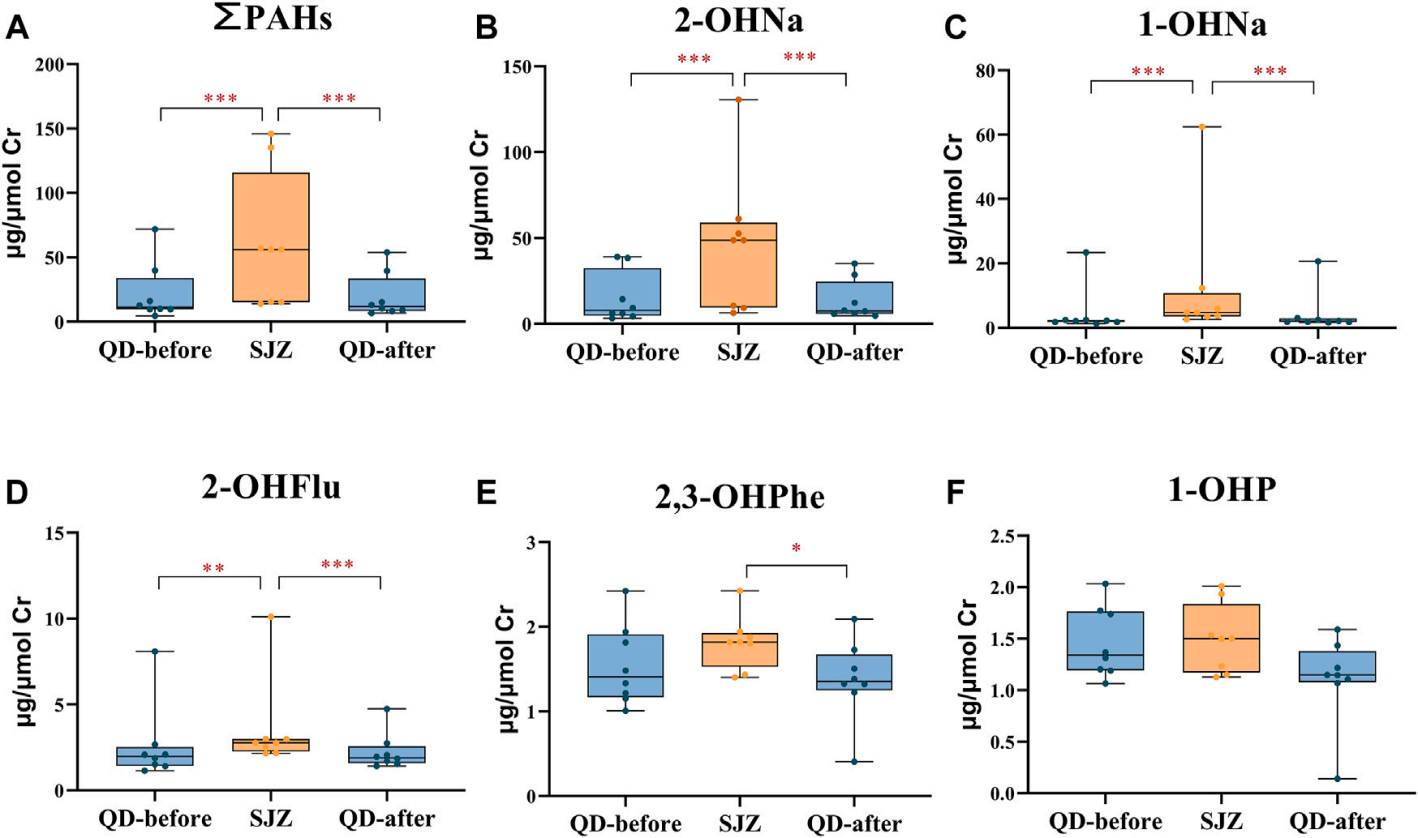
Urinary concentrations of polycyclic aromatic hydrocarbon metabolites in 8 participants in three periods. Yellow and blue solid dots represent samples from Shijiazhuang and Qingdao periods, horizontal lines in boxes represent the sample mean, and boxes represent the 25th–75th percentiles. *False discovery rate <5% after Benjamini–Hochberg adjustment. **False discovery rate <1% after Benjamini–Hochberg adjustment. ***False discovery rate <0.1% after Benjamini–Hochberg adjustment. **(A-F)** respectively represents the concentration of ∑PAHs, 2-OHNA, 1-OHNA, 2-OHflu, 2, 3-OHPHE and 1-OHP in the urine of participants in three periods.

The concentration in SJZ was significantly higher than in QD-before for ∑PAHs (390%), 2-OHNa (517%), 1-OHNa (121%), and 2-OHFlu (40%). The concentration of urinary PAHs in the QD-after period was significantly lower than in the SJZ period, and there were no significant differences between the QD-before period and the QD-after period (Figure 2).

### Changes in Platelet Parameters

**FIGURE 3:**
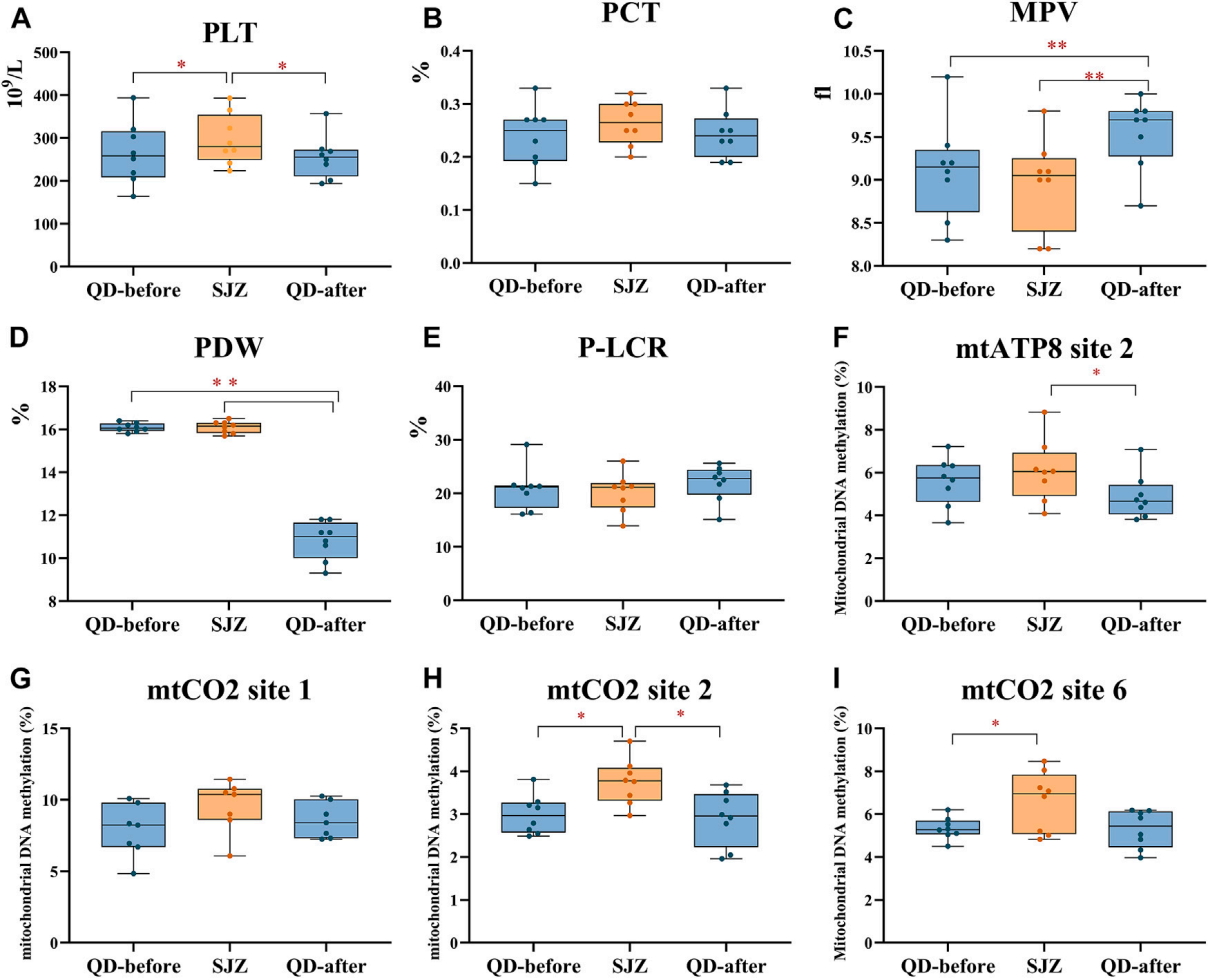
Thrombocyte parameters in 8 participants at three periods. Yellow and blue solid dots represent samples from Shijiazhuang and Qingdao periods, horizontal lines in boxes represent the sample median, and boxes represent the 25th–75th percentiles. *False discovery rate <5% after Benjamini–Hochberg adjustment. **False discovery rate <1% after Benjamini–Hochberg adjustment. **(A-E)** respectively represent the parameters of PLT, PCT, MPV, PDW, and P-LCR in the blood of participants at three periods. (F-I) successively represents the methylation levels of participants' platelets at mtATP8 site 2, mtCO2 site 1, mtCO2 site 2, and mtCO2 site 6 at the three stages.

Platelet count (PLT) in the SJZ period was 301.00 ± 62.54 × 10^9^/L, 12.7% higher than in the QD-before period (267.14 ± 78.52 × 10^9^/L). PLT in QD-after (258.00 ± 54.15 × 10^9^/L) was 14.3% lower than in SJZ after 6 days of recovery, and the difference also existed after subsequent multiple comparisons (Figure 3A). Mean platelet volume (MPV) was 0.44 fl higher at QD-after than at QD-before. However, it is worth noting that MPV decreased in SJZ (Figure 3C). Platelet distribution (PDW) in QD-after was 33% lower than that in QD-before (Figure 3D). The changes of platelet hematocrit (PCT) and platelet large cell ratio (P-LCR) were not significant in the three stages (Figures 3B,E).

Mitochondrial DNA methylation in each individual platelet was detected and observed by bisulfite PCR combined with pyrosequencing. Through the analysis of 13 methylation sites in 4 mitochondrial genes of participants’ platelets, the assessment presented that the methylation of *mtCO2 site 2* in the SJZ stage was higher than that in the QD-before stage (3.75 vs. 2.99%, *p* < 0.05) (Figure 3H). After 6 days of recovery, the participants’ *mtCO2 site 2* methylation was reduced (3.75 vs. 2.90%, *p* < 0.05). Similarly, the methylation of *mtATP8 site 2* increased at first and then decreased throughout the study period (5.59 vs. 6.08%, 6.08 vs. 4.89%, *p* > 0.05) (Figure 3F). The methylation of mtCO2 site 1 did not change significantly throughout the study period (Figure 3G). In addition, the *mtND5 site 1* methylation in SJZ (2.34%) was lower than that in QD-before (2.54%), and the methylation became higher after the recovery stage (2.01%) (Supplementary Figure S1G).

### Association Between Air Pollution and Platelet Parameters

**FIGURE 4:**
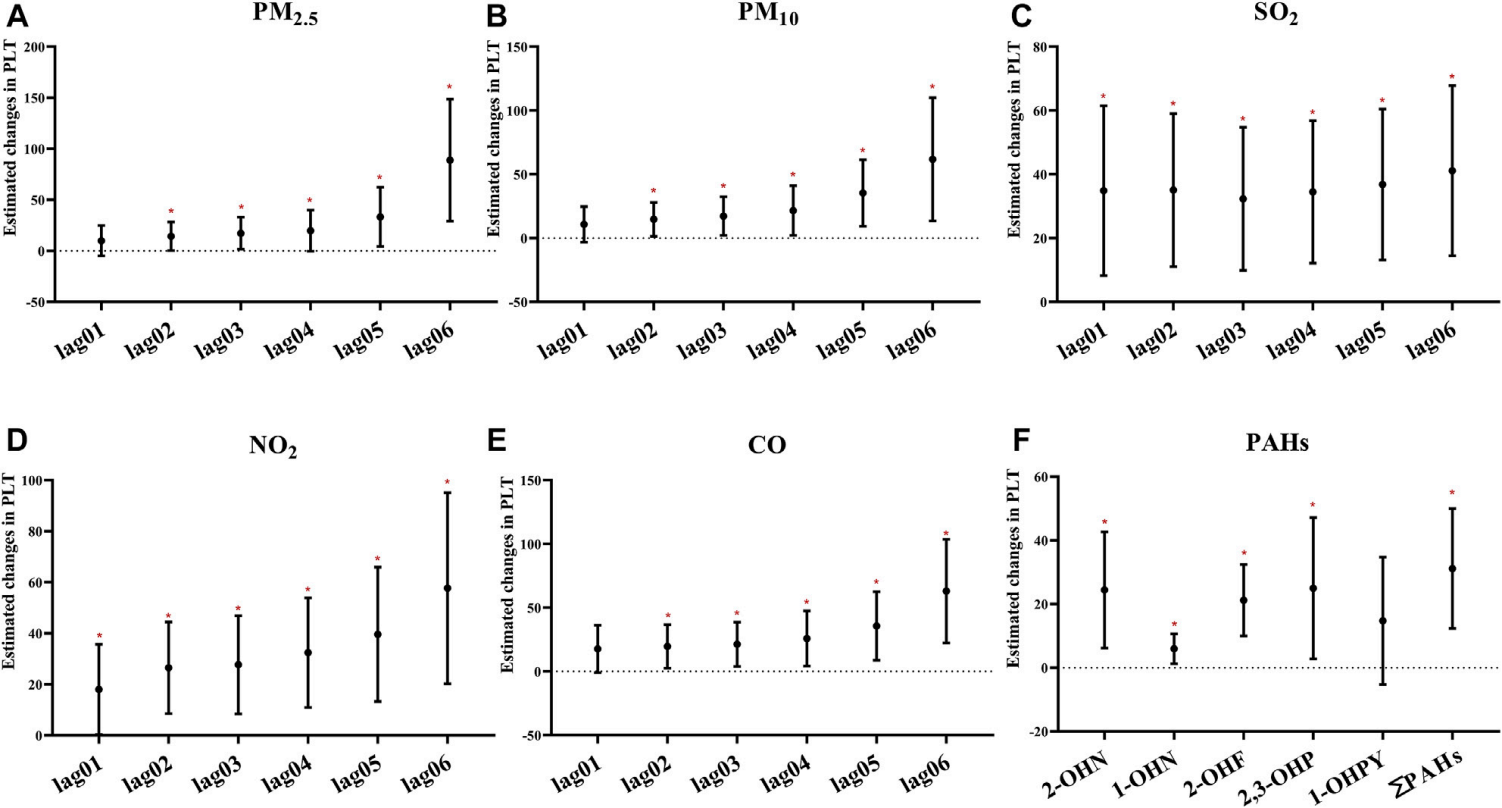
Estimated changes (95% confidence intervals) of PLT in the thrombocyte parameters. Changes (95% confidence intervals) in the biomarkers with IQR increases in air pollution indicators were calculated, respectively, at different time lags (days). **(A–E)** Indicates the situation of each pollutant separately. **(F)** Indicates the estimated changes (95% confidence intervals) in the thrombocyte parameters with IQR increases of PAHs. Models adjusted for age, BMI, height, and smoking status. Red * denotes *p* < 0.05.

Most air pollutants, PAHs, and PLT were positively correlated (Figure 4). In particular, the association based on the air pollution index (API) of 0–6 day moving averages (β_PM2.5_ = 88.90, 95% CI: 29.22–148.57; β_PM10_ = 61.83, 95% CI: 13.61, 110.05; β_SO2_ = 41.13, 95% CI: 14.46, 67.80; β_NO2_ = 57.70, 95% CI: 20.28, 95.12; β_CO_ = 62.99, 95% CI: 22.42,103.56, respectively, for an IQR increased) appeared to be stronger than those based on exposure metrics of 0–1 moving averages to 0–5 moving averages. These could indicate that cumulative exposure over multiple days may be able to capture the potential impact of air pollution more robustly than only 1 day. Additionally, 2-OHNa, 1-OHNa, 2-OHFlu, 2,3-OHPhe, and ∑PAHs were significantly associated with PLT in participants. Specifically, IQR increases in 2-OHNa (31.97 μg/μmol Cr) and 1-OHNa (2.84 μg/μmol Cr) were significantly associated with a 24.42 ×10^9^/L (95% CI: 6.18, 42.66) and 5.95 ×10^9^/L (95% CI: 1.25,10.64) increase in PLT (Figure 4F). Moreover, PLT increases by 21.17 ×10^9^/L (95% CI: 9.97, 32.37) for an IQR (1.07 μg/μmol Cr) along with increase in 2-OHFlu. An IQR increase in ∑PAHs (43.97 μg/μmol Cr) was significantly associated with a 31.15 ×10^9^/L (95% CI: 0.19, 1.01) increase in the *mtCO2* site 2 methylation.

**FIGURE 5:**
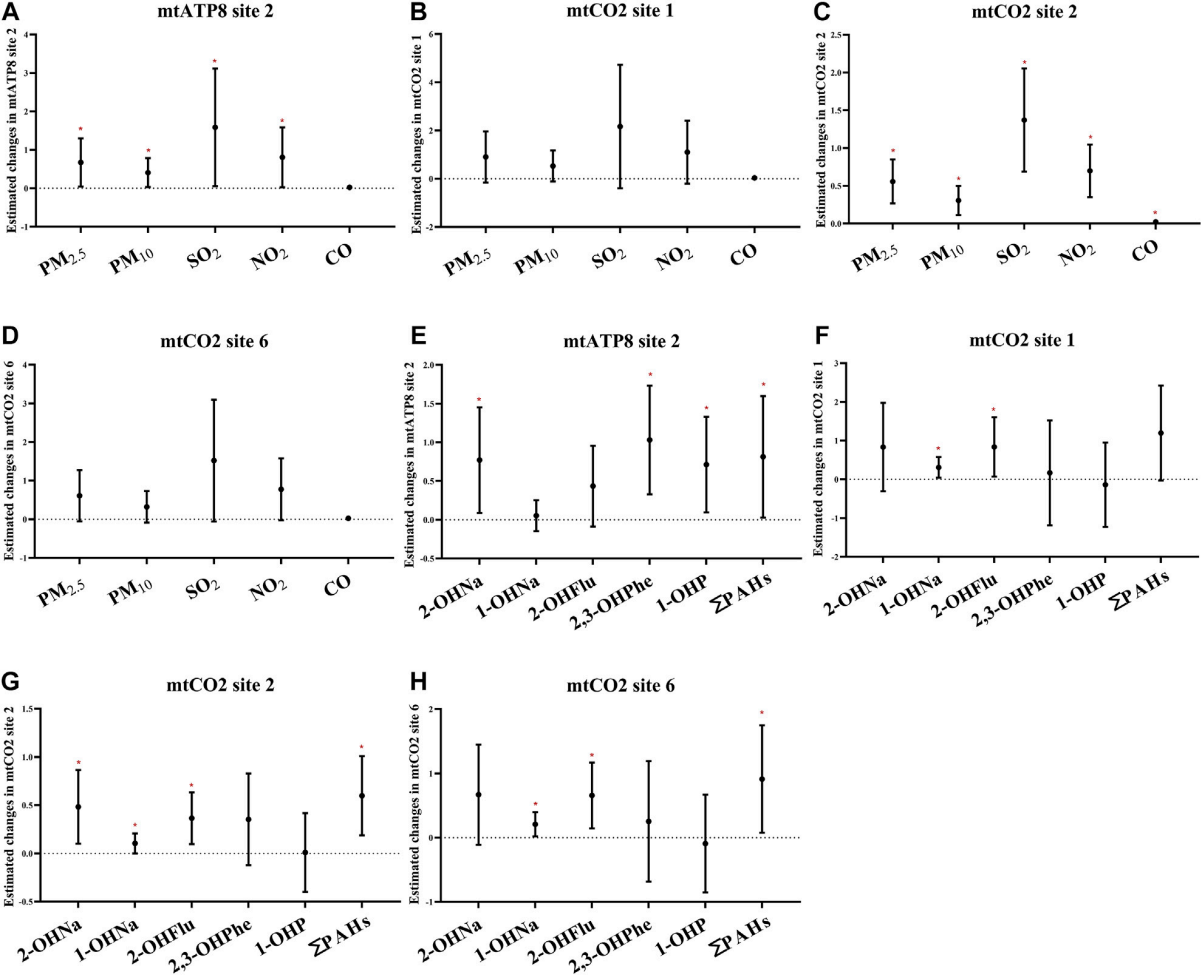
Estimated changes (95% confidence intervals) of platelet mitochondrial DNA methylation in the thrombocyte parameters. Changes (95% confidence intervals) in the biomarkers with IQR increases in air pollution indicators and PAHs were calculated. **(A–D)** Indicates the situation of each pollutant, separately. **(E–H)** Indicate the situation of PAHs. Models adjusted for age, BMI, height, and smoking status. * denotes *p* < 0.05.

**FIGURE 6:**
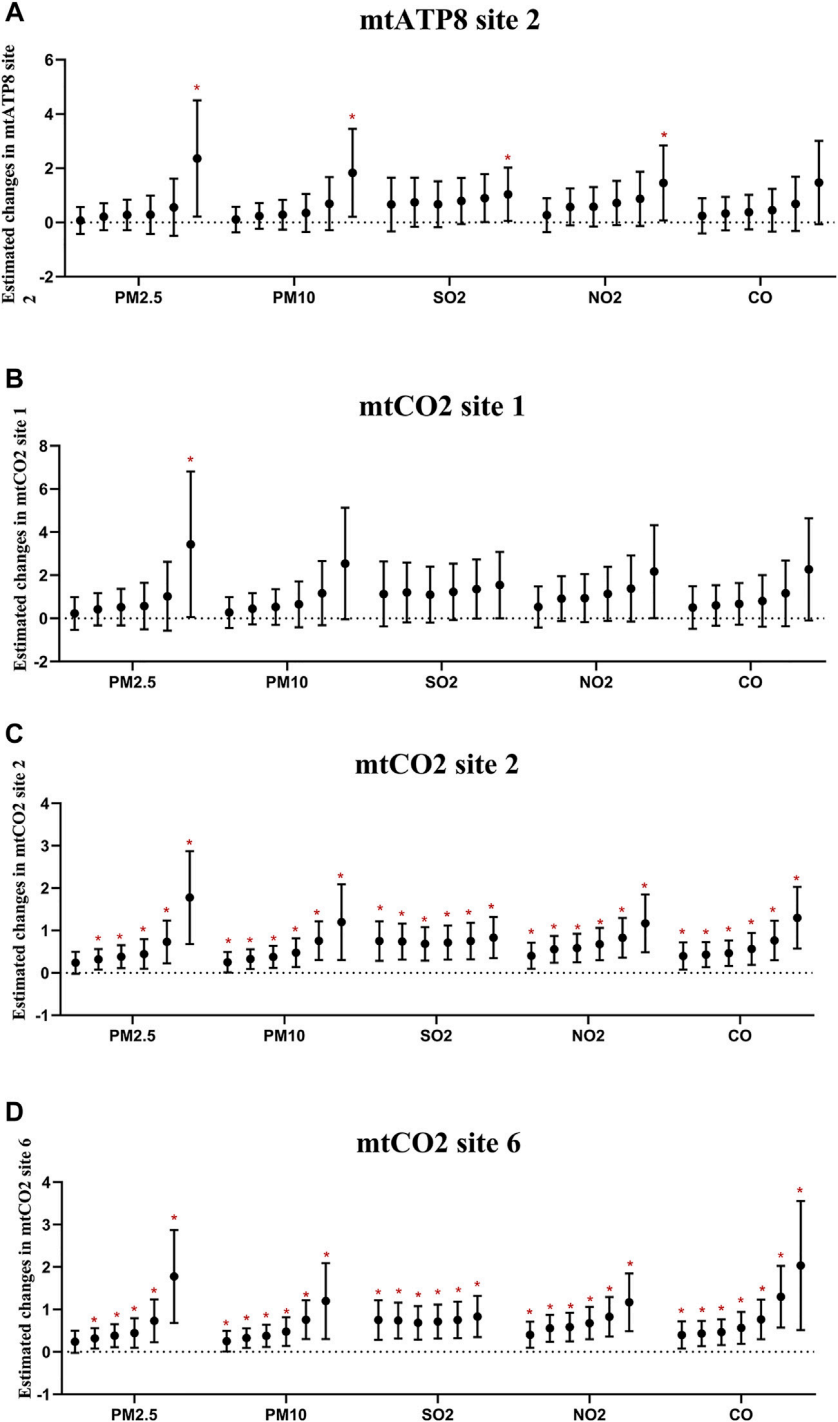
Estimated changes (95% confidence intervals) of platelet mitochondrial DNA methylation in the thrombocyte parameters. Changes (95% confidence intervals) in the biomarkers with IQR increases in air pollution indicators were calculated, respectively, at different time lags (days). **(A–D)** Indicate the situation of each gene. In each pollutant group, the cumulative lag from the left to the right is 1–6 days.

Furthermore, we performed the regression analyses to investigate the associations between air pollutants and selected methylation of mitochondrial CpG sites in platelets (Figure 5; Supplementary Figure S2). Through the analysis of the single pollutant model, the correlation between air pollutants and *mtATP8 site 2* was evaluated. It was found that there was a significant positive correlation between PM_2.5_, PM_10_, and *mtATP8*. (β_PM2.5_ = 0.67, 95% CI: 0.04,1.30, β_PM10_ = 0.41, 95% CI: 0.03,0.79, respectively, for an IQR increase, *P* values <0.05). In the same way, this positive correlation was also found in SO_2_ and NO_2_ with *mtATP8* (β_SO2_ = 1.59, 95% CI: 0.06,3.11; β_NO2_ = 0.81, 95% CI: 0.03, 1.59, respectively, for an IQR increased, all *p-*values <0.05). The *mtCO2 site 2* methylation is positively correlated with the PM_2.5_ and PM_10_ (β_PM2.5_ = 0.47, 95% CI: 0.18,0.76, β_PM10_ = 0.25, 95% CI: 0.06,0.44, respectively, for an IQR increase, *p-*values <0.05). Subsequently, the same positive association was identified in SO_2_, NO_2_, and CO (β_SO2_ = 1.16, 95% CI: 0.48,1.85, β_NO2_ = 0.59, 95% CI: 0.25,0.94, β_co_ = 0.02, 95% CI: 0.01,0.03, respectively, for an IQR increase, all *p-*values <0.05). On the other hand, an IQR increase in CO was significantly associated with a 0.33% (95% CI: −0.52, −0.15) decrease in *mtCO2 site 2* methylation. We diagnosed significant positive correlations that were seen between 2-OHNa and 1-OHNa with *mtCO2 site* 2 methylation (Figure 5). The methylation of *mtCO2 site 2* increased by 0.48 (95% CI: 0.09, 0.87) and 0.1 (95% CI: 0.00,0.21) with each increase in IQR for 2-OHN (31.97 μg/μmol Cr) and 1-OHN (2.84 μg/μmol Cr). *mtCO2 site 2* methylation increases by 0.36 (95% CI: 0.10, 0.63) for an IQR (1.07 μg/μmol Cr) increase in 2-OHFlu. An IQR increase in ∑PAHs (43.97 μg/μmol Cr) was significantly associated with a 0.58% (95% CI: 0.19, 1.01) increase in *mtCO2 site 2* methylation. There was a positive correlation between 0- to 6-day moving averages of the air pollution index and platelet mitochondrial DNA methylation at mtATP8 site 2 (β_PM2.5_ = 2.40, 95% CI: 0.21,4.50; β_PM10_ = 1.83, 95% CI: 0.21,3.44; β_SO2_ = 1.04, 95% CI: 0.05, 2.00; β_NO2_ = 1.46, 95% CI: 0.08, 2.83, respectively, for an IQR increased). For *mtCO2* site 2 and site 6, the association based on the air pollution index (API) of 0- to 6-day moving averages appeared to be stronger than those based on exposure metrics of 0–1 moving averages to 0–5 moving averages (Figure 6).

In the sensitivity analysis, which assessed the correlation between excluding smoking participants, no difference was found in *mtCO2 site2* methylation and PLT (Supplementary Figures S3, S4). However, smoking was found to attenuate the association between urinary PAH metabolites and PLT, except for 2-OHNa, 2-OHFlu, and ∑PAH metabolites.

## Discussion

**FIGURE 7:**
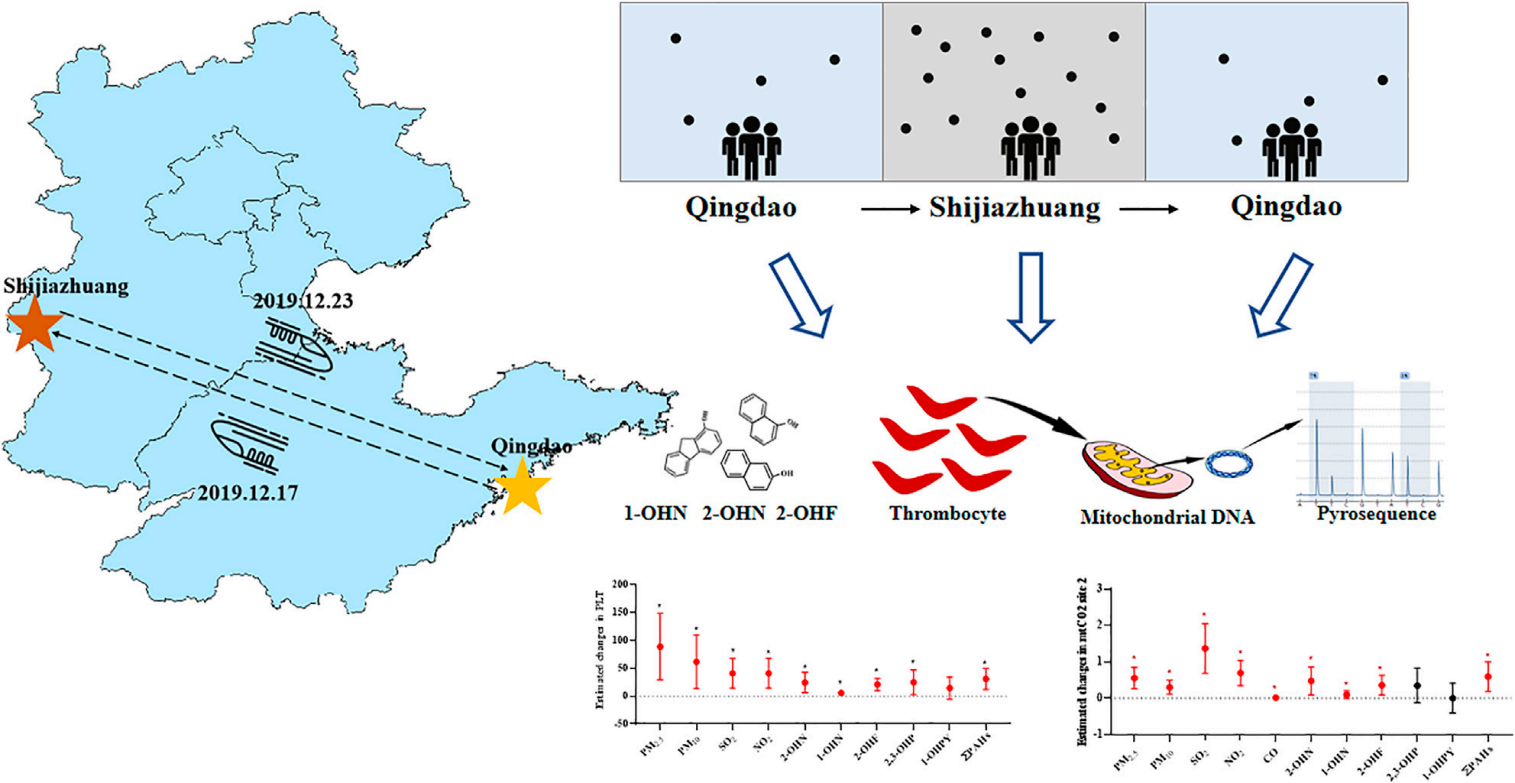
Flow chart of the panel study.

In our research, a prospective panel study was conducted to track a group of healthy young people who travelled from Qingdao to Shijiazhuang for 6 days. Investigating the differences in air quality involved two places to identify new and more sensitive biomarkers after acute exposure to air pollution. This panel study revealed that PLT and mitochondrial DNA methylation in platelets was associated with acute air pollution exposure (Figure 7). Panel study is a kind of environmental epidemiology research method. The effects of various confounding factors on experimental results were reduced by comparing the participants’ own before and after indicators. At the same time, longitudinal measurement and recording of time indicators at multiple nodes can accurately grasp the time effect relationship between exposure and health outcomes of research objects, so as to reveal the mechanism of the impact of pollutants on health. This method, due to the research on the human health effects of exposure to air pollution in many advantages, is widely used (Kipen et al., 2010; Bai et al., 2019).

Participants in SJZ appeared to have a higher level of PAH metabolites in their urine, most likely due to the atmospheric particles in SJZ containing more polycyclic aromatic hydrocarbons. PAHs are important components of atmospheric aerosols, which are mostly concentrated in fine particles (Li et al., 2016; He et al., 2018). Urinary PAHs are frequently used to estimate total exposure to PAHs (He et al., 2021). Previous studies have shown that the concentration of PAHs in the air of China is high inland and low along the coast (Li et al., 2014).

The platelet count of the participants increased after acute exposure to air pollution and decreased to the original level after recovery. After being associated with pollutants and PAH metabolites, it was found that the platelet count of the participants increased with the increase in API and PAH, which is consistent with the results of previous studies (Emmerechts et al., 2012; Zhang et al., 2018; Dai et al., 2019). A cohort study of 362,396 adults in Taiwan showed that every 10 μg/m^3^ increase in PM_2.5_ was associated with a 0.42 (95% CI: 0.38%, 0.47%) and 0.49% (95% CI: 0.44%, 0.54%) increase in platelet count in men and women, respectively (Zhang et al., 2018). Air pollution might induce platelet aggregation, increasing number and activation through inflammation and oxidative stress, vascular endothelial injury, or other nervous system imbalance (Brook et al., 2010; Hamanaka and Mutlu, 2018). It was also revealed that PLT of participants decreased to the QD-before level after the recovery period (Figure 3A), which may be closely related to the life cycle of platelets (Yeung et al., 2018). As a kind of anucleated cell, mature platelets are unable to divide and proliferate and generally survive for 5–7 days after they are separated from megakaryocytes (Holinstat, 2017). Platelet size (specifically MPV and P-LCR) is the primary determinant of platelet activity. Although results of MPV were delayed (Figure 3C), our results are consistent with previous studies that acute exposure to pollutants increases MPV in healthy subjects (Yarlioglues et al., 2012). One possible biological explanation is that considering the reason, large platelets contain more alpha particles, which can produce and release more thromboxane A2 and P-selectin, and increase the binding with fibrinogen which leads to enhanced thrombogenesis (Holinstat, 2017). During its normal life cycle, platelets decrease in size such that young platelets are measurably larger than older platelets (van der Meijden and Heemskerk, 2019). Yuan et al. found that elevated MPV contributed 13.6% to the relationship between PAH exposure and 10-year ASCVD risk (Hu et al., 2018). In clinical application, MPV and P-LCR can be the tool for predicting myocardial infarction, complications after coronary intervention, and death of coronary heart disease (Tian et al., 2018). However, changes in platelet size in the general population after undergoing acute air pollution have not been studied. In addition, our study also analyzed other platelet parameters and found that platelet count can also be a sensitive biomarker of acute air pollution. In general, the results show that acute exposure to air pollution can increase MPV and PLT in the general population, increasing the risk of cardiovascular events.

As a kind of non-nucleated cell, platelet mitochondrial DNA plays a crucial role in cell regulation. Epigenetic changes in genes, such as elevated levels of methylation, are associated with an increased risk of cardiovascular disease (Roy et al., 2019). Usually, mitochondrial genomic DNA methylation levels are shallow. Before and after QD, we found low mtDNA methylation levels in platelets but high mtDNA methylation levels during SJZ. Hypermethylation of platelet mtDNA can affect its metabolism, activation, and apoptosis. Mitochondrial damage or dysfunction, as observed in some diseases, notably results in significantly reduced platelet survival and an increased risk of thrombotic events (Bauereisen et al., 1964; Melchinger et al., 2019). So this may be why air pollution raises the risk of cardiovascular disease. Previous studies have focused on the methylation of mitochondrial genes in peripheral blood leukocytes, but there is a lack of studies on the relationship between the methylation of mitochondrial genes in a single type of cell and acute air pollution (Byun et al., 2013; Wang et al., 2020a). The *mtCO2* site two methylations of participants in the SJZ period were significantly higher than that in other periods. COX is a component of the respiratory electron transport chain and consists of 1–3 subunits (*mtCO1*, *mtCO2*, and *mtCO3*) that form the functional core of complex IV. In addition, mutations in the *COX* gene have been linked to metabolic disorders, mainly affecting high-energy tissues. Among many mitochondrial diseases, the COX dysfunction complex is considered to be the most serious. Recent studies have shown that the lung is vital for platelet biogenesis (Mallah et al., 2020). Previous studies have confirmed that cigarette smoking could induce platelet aggregation and reduce the ability of the platelet mitochondrial electron transport system. This seems to provide a plausible explanation for the study: air pollution leads to increased *mtCO2* methylation and decreased capacity of platelet mitochondrial electron transport systems, resulting in changes in participants’ blood indicators.

The mammalian ATP8 encodes a part of the ATP synthase F0 subunit structure in the mitochondrial genome. This mitochondrial gene is one of the mitochondrial inner respiratory chain genes (Martin and Fry, 2018). Chen et al. found that methylation was positively correlated with urinary 1-hydroxypyrene levels (*β* = 0.029, 95 CI%: 0.01, 0.048) (He et al., 2019). Numerous other studies have shown that air pollution and polycyclic aromatic hydrocarbon (PAH) pollution are associated with nuclear DNA methylation (Li et al., 2018b; He et al., 2019). Still, the relationship between PAHs and platelet mitochondrial DNA methylation has not been studied yet. Our results revealed that the methylation level of *mtCO2* increased with the increase in the pollutant level that complemented the previous study (Errichiello et al., 2015). Despite the lack of direct evidence, a large number of epidemiological studies have shown that mitochondrial DNA is more susceptible to pollution than nuclear DNA, resulting in methylation (Shukla et al., 2003), which may affect mitochondrial gene expression, biogenesis, cell function (Iacobazzi et al., 2013), and interaction with air pollution to increase the risk of CVD. Mitochondrial DNA methylation has been proposed as a biomarker and diagnostic tool for the next generation. As a clinically readily available substance, platelet mitochondrial methylation has excellent potential as a new biomarker. However, changes in platelet size in the general population after acute contamination have not been studied. In addition, our study also analyzed other platelet parameters and found that platelet count can also be a sensitive biomarker of acute air pollution.

The strengths of this research are described in the following. First, various platelet markers were measured, including PLT, MPV, PDW, PCT, and P-LCR, whereas most studies have only measured PLT. Second, this research, unlike previous studies, chose the platelet mtDNA as the research object. It provides more evidence to reveal the link between air pollution and cardiovascular disease. Third, the recovery period of participants was set to investigate whether the platelet changes caused by short-term environmental pollution are reversible. Finally, pyrosequencing, the gold standard of epigenetics, was used to detect the methylation status of specific sites, which improved the reliability of the results. However, several limitations still existed in the current study. First, this natural experiment cannot control all factors, like diet and circadian rhythm, which could influence the outcomes under study. Second, the study included only eight participants in all three phases, although statistically significant differences were found. However, the number of samples may affect the accuracy of the results. Finally, no wearable devices were used to monitor the pollution exposure dose of each participant. Thus, it was impossible to assess individual exposure accurately.

## Conclusion

In summary, this study suggests that acute exposure to air pollution can induce changes in platelet parameters. Platelet count and mitochondrial DNA methylation of *mtCO2 site2* in platelets from healthy subjects may be sensitive biomarkers for acute air pollution exposure. The platelet mitochondrial DNA methylation changes may provide a preliminary understanding of the epigenetic regulatory pattern of platelet parameter changes caused by acute air pollution exposure.

## Data Availability

The original contributions presented in the study are included in the article/Supplementary Material, further inquiries can be directed to the corresponding authors.
