# Supplementary material for: Platelet Mitochondrial DNA Methylation as Epigenetic Biomarker of Short-Term Air Pollution Exposure in Healthy Subjects
Source: Front Mol Biosci. 2022 Jan 19;8:803488. doi: 10.3389/fmolb.2021.803488 (PMC8809460; doi:10.3389/fmolb.2021.803488)
Supplement: Supplementary file 1 [file DataSheet1.DOCX]

**Platelet mitochondrial DNA methylation as epigenetic biomarker of short-term air pollution exposure in healthy subjects**

Huimin Sun^a,1^, Yanting Li^a,1^, Jianzhong Zhang^a^, Teng Shi^a^, Xin Li^a^, Xue Cao^a^, Tao Wang^a^, Nan Kong^a^, Yaxian Pang^b^, Tsendmaa Bold^a^, Yuxin Zheng^a^, Rong Zhang^b,^* Jinglong Tang^a,^*

a Department of Occupational and Environmental Health, School of Public Health, Qingdao University, Qingdao, China, 266071.

b Department of Toxicology, School of Public Health, Hebei Medical University, Shijiazhuang, China, 050017.

^1^ These author contributed equally to this work.

*Corresponding author: rongzhang@hebmu.edu.cn (Rong Zhang) or tangjinglong@qdu.edu.cn (Jinglong Tang)

**Supplemental Table 1.** Air quality index of Shijiazhuang and Qingdao in three study periods

| Stage | API | Mean | SD | Median | Q1 | Q3 | IQR |
| --- | --- | --- | --- | --- | --- | --- | --- |
| QD-before | PM_2.5_ | 31.93 | 14.43 | 32.79 | 18.38 | 40.71 | 22.33 |
|  | PM_10_ | 58.81 | 21.55 | 65.27 | 33.67 | 77.46 | 43.79 |
|  | SO_2_ | 7.70 | 3.15 | 7.40 | 4.71 | 10.00 | 5.29 |
|  | NO_2_ | 41.06 | 9.26 | 41.42 | 32.38 | 44.13 | 11.75 |
|  | CO | 0.70 | 0.15 | 0.71 | 0.58 | 0.83 | 0.26 |
| SJZ | PM_2.5_ | 76.25 | 42.08 | 74.19 | 43.46 | 105.29 | 61.83 |
|  | PM_10_ | 118.04 | 58.04 | 120.42 | 74.50 | 155.33 | 80.83 |
|  | SO_2_ | 15.59 | 2.70 | 15.56 | 14.00 | 17.87 | 3.87 |
|  | NO_2_ | 61.04 | 13.51 | 63.69 | 52.75 | 70.08 | 17.33 |
|  | CO | 1.40 | 0.58 | 1.45 | 0.97 | 1.83 | 0.86 |
| QD-after | PM_2.5_ | 57.50 | 13.27 | 54.31 | 48.42 | 61.50 | 13.08 |
|  | PM_10_ | 87.10 | 19.50 | 85.10 | 69.75 | 101.21 | 31.46 |
|  | SO_2_ | 7.96 | 1.60 | 7.85 | 6.79 | 9.08 | 2.29 |
|  | NO_2_ | 46.05 | 7.90 | 48.17 | 40.38 | 50.42 | 10.04 |
|  | CO | 0.96 | 0.16 | 0.93 | 0.85 | 1.14 | 0.29 |

Deﬁnition of abbreviations: API = air pollution indicators, PM_2.5_ = fine particulate matter (μg/m^3^), PM_10_ = [inhalable](javascript:;) particulate matter (μg/m^3^), SO_2_ = sulfur dioxide (μg/m^3^), NO_2_ = nitrogen dioxide (μg/m^3^), CO = [carbon](javascript:;) [monoxide](javascript:;) (mg/m^3^), Q1 = lower quartile, Q3 = upper quartile, SD = standard deviation, IQR = interquartile range

**Supplemental Table 2.** Spearman’s correlation coefficient among seven air pollution indicators^a^

| API | PM_2.5_ | PM_10_ | SO_2_ | NO_2_ | CO |
| --- | --- | --- | --- | --- | --- |
| PM_2.5_ | 1.00 | 0.98* | 0.58* | 0.81* | 0.97* |
| PM_10_ |  | 1.00 | 0.67* | 0.87* | 0.97* |
| SO_2_ |  |  | 1.00* | 0.80* | 0.72* |
| NO_2_ |  |  |  | 1.00 | 0.87* |
| CO |  |  |  |  | 1.00 |

Deﬁnition of abbreviations: API = air pollution indicators, PM_2.5_ = fine particulate matter (μg/m^3^), PM_10_ = [inhalable](javascript:;) particulate matter (μg/m^3^), SO_2_ = sulfur dioxide (μg/m^3^), NO_2_ = nitrogen dioxide (μg/m^3^), CO = [carbon](javascript:;) [monoxide](javascript:;) (mg/m^3^)

^a^Spearman correlation coefficients were calculated for pair wise correlation analyses between seven air pollution indicators. The statistically significant values presented as * *P*＜0.05 in the test on correlation coefficient.


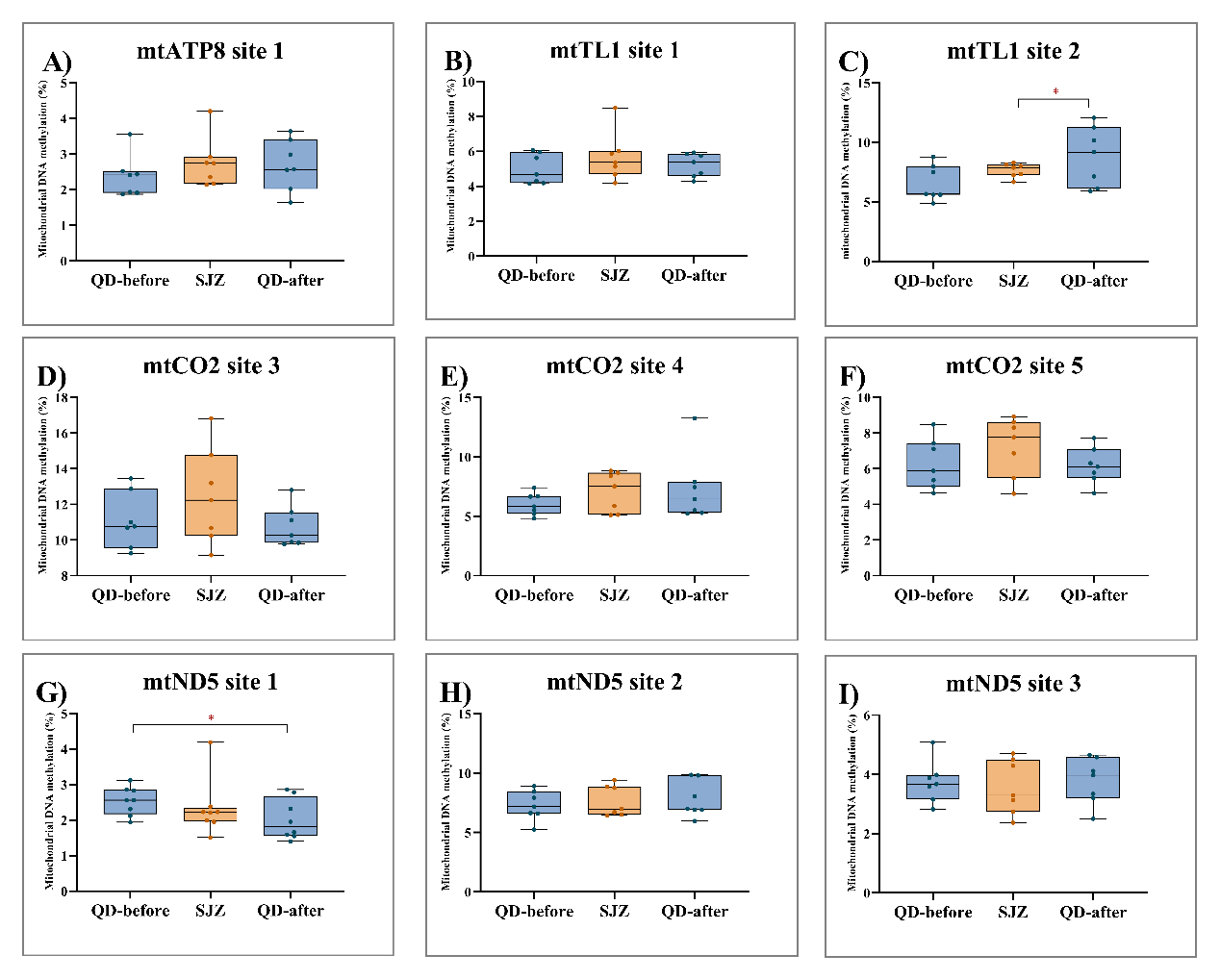


**Supplemental Figure 1.** Platelet mitochondrial DNA methylation in 8 participants at three periods. Yellow and blue solid dots represent samples from Shijiazhuang and Qingdao periods, horizontal lines in boxes represent sample mean, and boxes represent 25th - 75th percentiles.

*False discovery rate <5%;

**False discovery rate <1%;


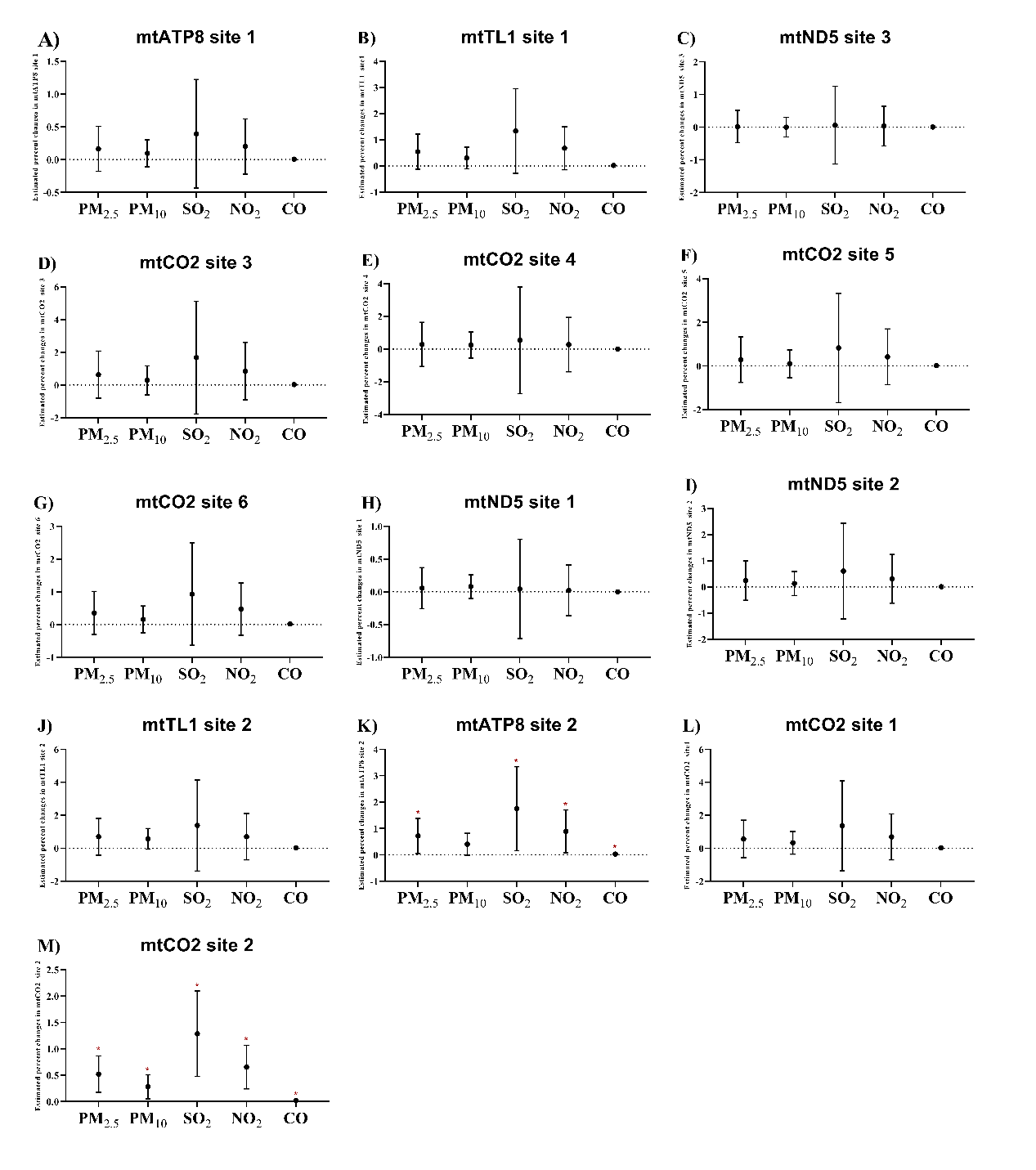
**Supplemental Figure 2.** Estimated changes (95% confidence intervals) in the thrombocyte parameters. Changes (95% confidence intervals) in the biomarkers with IQR increases in air pollution indicators were calculated. Models adjusted for age, BMI, height and smoking status. * denotes *P* < 0.05.


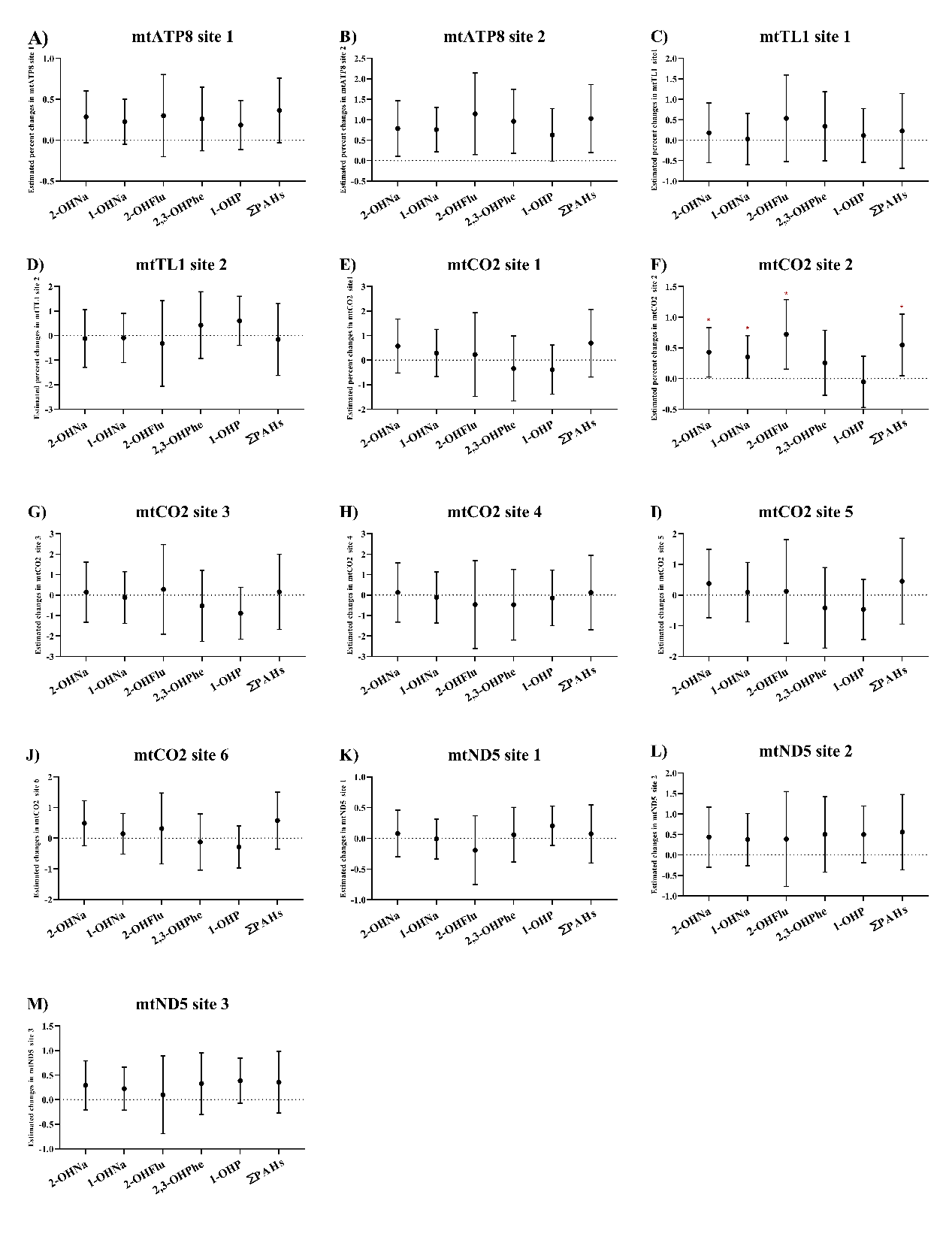
**Supplemental Figure 3.** The estimated changes (95% confidence intervals) of methylation sites associated with quartiles of each air pollutant, estimated by the mix model (excluding smoking participants). Model adjusted for age, BMI, height. * denotes *P* < 0.05.


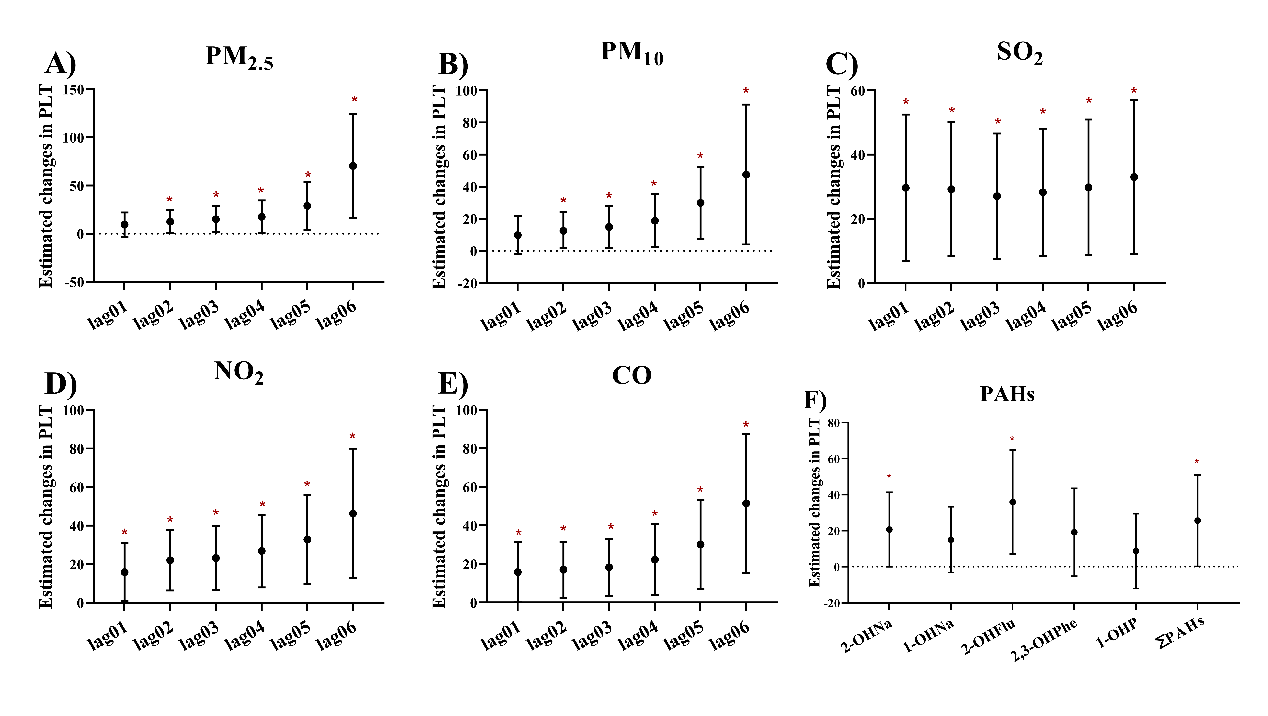
**Supplemental Figure 4.** Excluding participants who smoked, the mixed models estimated that the estimated changes of PLT (95% confidence intervals) were associated with the quartile of each urinary PAH metabolite Model adjusted for age, BMI, height. * denotes *P* < 0.05.
